# How Clinicians Decide? Exploring Complexity of Antibiotic Prescribing in Emergency Departments Using Video-Reflexive Ethnography

**DOI:** 10.1177/10497323231198144

**Published:** 2023-10-23

**Authors:** Mila Obucina, Laura Hamill, Ronald Huynh, Kylie Alcorn, Jack Cross, Amy Sweeny, Gerben Keijzers

**Affiliations:** 1 Department of Emergency Medicine, 3556 Gold Coast Health, Gold Coast, QLD, Australia; 2 School of Medicine, 97562 Griffith University, Gold Coast, QLD, Australia; 3 63588 Canterbury Health DHB, Christchurch, New Zealand; 4 Bond University, Gold Coast, QLD, Australia

**Keywords:** emergency dependent, antibiotic prescribing, decision-support tools, ethnography, video-reflexivity

## Abstract

Antibiotic overprescribing is a global issue that significantly contributes to increased antimicrobial resistance. Strengthening antimicrobial prescribing practices should be considered a priority. The emergency department (ED) represents a setting where antibiotics are frequently prescribed, but the determinants that influence prescribing choices are complex and multifaceted. We conducted an exploratory qualitative study to investigate the contextual factors that influence antibiotic prescribing choices among clinicians in the ED. The study employed video-reflexive ethnography (VRE) to capture prospective clinical decision-making in situated practice. Data collection involved fieldwork observations, video observations, and delivery of facilitated group reflexive sessions, where clinicians viewed a selection of recorded video snippets relating to antibiotic prescribing. Study was conducted across two EDs within the same health service in Australia. A total of 29 clinical conversations focusing on antibiotic prescribing were recorded. Additionally, 34 clinicians participated in group reflexive sessions. Thematic analysis from the transcribed data yielded four themes: ‘importance of clinical judgment’, ‘usability of prescribing guidelines’, ‘managing patient expectations’, and ‘context-dependent disruptions’. Our findings provide insights into the challenges faced by clinicians in navigating complex ED environment, utilising electronic decision-support tools and engaging in discussions about patient treatments with senior clinicians. The findings also indicate that VRE is useful in visualising full complexity of the ED setting, and in initiating meaningful discussions among clinical teams. Integrating the use of VRE in everyday clinical settings can potentially facilitate the implementation of pragmatic solutions for delivering effective antibiotic stewardship practices.

## Introduction

Antimicrobial resistance has been recognised as a significant global concern. Antibiotic overprescribing contributes to reduced medication efficacy, heightened risk of severe illnesses, and further disease spread among populations (WHO, 2021). In response to this rising issue, healthcare agencies have called for more effective implementation of antimicrobial stewardship practices, namely, targeted initiatives that improve how antibiotics are prescribed by clinicians. Driven by compelling scholarly evidence, researchers and clinicians alike have redoubled efforts to improve antibiotic prescribing and promote their wise and rational use (Church & McKillip, 2021; Malmgren et al., 2019; Mobarki et al., 2019; Schwartz et al., 2021). Such collective efforts aim to ensure the preservation of antibiotic effectiveness for future generations.

The emergency department (ED) is one clinical setting where antibiotics are commonly prescribed. EDs are dynamic and fast-paced, often described as complex, chaotic, and challenging environments (Pulia et al., 2018, 2020), where high-stake decisions about patient treatments are frequently made within tight timeframes and limited resources (Press et al., 2015). Most healthcare organisations have evidence-based clinical guidelines to assist in selection of suitable antibiotic treatments, including recommendations on appropriate antibiotic type, optimal dose, administering route, and treatment duration. Previous research addressing overuse and misuse of antibiotics in the ED setting is well documented; however, numerous challenges persist despite efforts to improve practice (Charani & Holmes, 2019; May et al., 2021).

Scholars suggest that implementation of research into real-world hospital settings is challenging due to the inherent complexity of the healthcare environment (Braithwaite et al., 2017; Greenhalgh & Papoutsi, 2018). For instance, a recent systematic review emphasised that successful implementation of healthcare interventions should not be solely reliant on predetermined strategies but also on the awareness of influences that arise from the intricate complexity of the hospital system (Geerligs et al., 2018). Similarly, the use of reductionist approaches which distil the complexness of clinical practice often fall short, leading to missed opportunities for in-depth comprehension of the influence of complexity on implementation (Churruca et al., 2019; Rapport et al., 2022) These findings highlight the ongoing difficulties in research translation and underscore the need for novel approaches that recognise and address the complexity of healthcare environments.

### Use of Video-Ethnographic Methods in Healthcare

Increasingly, the use of ethnographic methods to aid research implementation has been proposed, highlighting their potential to “capture context-specific phenomena, understand insiders’ perspectives, and study complex interactions” (Gertner et al., 2021, p. 8). Ethnographic observations, including video-ethnographic observations, provide a valuable contextual understanding of how individuals interpret their surroundings and how this shapes their behaviour and actions (Asan & Montague, 2014; Dixon-Woods, 2010). Such methods offer a unique way of ‘visualising’ real-life clinical practice by fully embracing rather than attempting to simplify healthcare’s inherent complexity (Carroll, 2009; Iedema, 2019; Pink, 2001). Thus, intricate aspects of everyday work practices are placed into the focus of observation, enabling insights into diverse perspectives (Slutskaya et al., 2018).

Video-reflexive ethnography (VRE) is one such method that has predominantly been used to understand, interpret, and evaluate clinical practice (Carroll & Mesman, 2018; Iedema et al., 2018). In its simplest form, VRE involves *video-ethnography*, or filming of ‘work that people do’ in clinical practice, and *video-reflexivity*, which includes playing back selected video snippets in group sessions to prompt collaborative interpretation and discussion. Clinicians subsequently examine and reassess their own interactions and behaviours, with opportunities to analytically problem-solve, learn, and offer potential solutions to known practice challenges (Iedema & Xyrichis, 2021). A description of a typical VRE process is included in Table 1.

**Table 1.** Video-Reflexive Ethnography – Application in Practice.

	Application in practice
Phase	Description
Phase 1	The first stage of a typical VRE research project often involves ethnographic field observations. The researcher immerses themselves in clinical settings to become familiar with local processes, gain an understanding of workflow and team dynamics, identify potential practices that should be recorded, establish relationships with relevant stakeholders, and engage in conversations and informal interviews with clinical staff.
Phase 2	During the video-observation phase of a VRE research project, the researcher records a predetermined task, action, or phenomenon of interest as it unfolds in a clinical setting. To help guide considerations of what to capture on video, the researcher creatively applies VRE theoretical principles to situations at hand and consults various clinicians working in the space. As a result, certain aspects of practice are captured on film, either in a planned or opportunistic fashion, guided by insights from participants on what they consider important, interesting, or in need of improvement.
Phase 3	Video-reflexivity is the final phase of a VRE research project. Most commonly, the researcher selects suitable video clips that are reflective of the healthcare practice. At times, the researcher engages in consultations with senior clinicians to seek guidance on what is deemed appropriate and most likely to generate positive outcomes, rather than cause controversy, disagreements, or negativity. Selected snippets are then used to facilitate group sessions, where collective discussion, team learning, and new insights about practice occur. This final phase is a two-step process. **3.1** Firstly, the researcher must engage in a process of careful deliberation to determine what to include or exclude from the video and determine the appropriate amount of footage to present, as this is rarely a self-evident process. The selection of video clips is often linked to the key research aims and made in consultation with the research team or clinicians. Practical considerations, such as the quality of the recording, time restraints, availability of clinicians to participate in group sessions, and the suitability of the space, all influence the preparation and delivery choices made by the researcher. **3.2** Secondly, the researcher delivers group reflexive sessions to show video clips and facilitate collective discussion and deliberation regarding observed work practices. Group sessions provide participants with an opportunity to make sense of the aspects of practice captured on video and identify habits, assumptions, or procedures that may require modification. As a result of collective reflections, teams may consider adjusting protocols or implementing practices with higher levels of appropriateness and effectiveness, by doing things more systematically or more consistently, enabling practice change to occur.

Adapted from Iedema, R., Carroll, K., Collier, A., Hor, S. Y., Mesman, J., & Wyer, M. (2018). *Video-reflexive ethnography in health research and healthcare improvement: Theory and application*. CRC Press.

There are four theoretical principles guiding the application of VRE – exnovation, collaboration, reflexivity, and care (Iedema et al., 2018). This method is based on the perspective that initiating change in real-world setting should always begin with ‘exnovation’, a term used to describe ‘innovation that comes from within’ (Iedema et al., 2013). VRE further recognises that clinicians and patients hold the closest proximity to care and should play an active role in research co-creation. Moreover, reflexivity in VRE prompts collaborative discussion and unpacking of routine practices, behaviours, and habits that become ‘visible’ through use of video. Finally, establishing care in VRE ensures that participants experience a sense of ‘psychological safety’, enabling them to engage in open discussion about their own and colleagues’ practices, despite the inherent discomfort of being filmed.

Drawing on classical ethnographic principles, VRE endeavours to provide rich, in-depth accounts of ‘lived experiences’ of participants in their natural setting, based on the long-term observations by the researcher. In classic ethnography, however, a researcher fundamentally uses a camera to collect observable data and produce a well-polished ethnographic footage that conveys specific anthropological knowledge (Ruby, 2006). On the other hand, VRE uses video to generate reflexive conversation among clinicians about their own practices so that collective learning and change can occur (Iedema et al., 2018). As a result, clinicians are afforded the opportunity to tangibly observe the nature of their work as is ‘routinely done’, revealing why they do things in a particular way (Iedema & Xyrichis, 2021). This unique form of reflexivity aims to trigger rethinking of what clinicians know, what they assume, and what they do, opening the way for the transformative change to happen (Carroll et al., 2021; Manojlovich et al., 2019).

Use of video-recorded materials in healthcare research is not a new phenomenon (Asan & Montague, 2014; Iedema et al., 2006; Parry et al., 2016). However, the use of video-ethnographic research remains underutilised in healthcare practice, despite strong indications that it can improve clinical practice (Bezemer et al., 2017; Cattaneo et al., 2020). For instance, VRE has previously been used to explore clinical communication in highly complex settings such as intensive care (Carroll et al., 2008), neonatal intensive care (Pedersen & Mesman, 2021), and ambulance-to-ED clinical handovers (Iedema et al., 2012). Similarly, patients’ experience of receiving care was examined by use of VRE in dementia care (Hung et al., 2018), palliative care (Collier et al., 2019), and infection prevention and control (Collier & Wyer, 2016; Wyer et al., 2017).

Building on our previous research on challenges and potential improvements to antibiotic prescribing in EDs (Denny et al., 2019; Hamill et al., 2021), we sought to examine clinical communication around antibiotic treatment decisions as they unfolded at the point of care. Furthermore, as no prior studies used VRE to explore antibiotic prescribing practices, we assessed the acceptability of video-reflexive methods among clinicians working in a complex ED environment. Specifically, we sought to answer the following research questions:

1) What factors shape routine clinical decision-making on antibiotic prescribing in EDs?
2) Is VRE considered acceptable as an improvement tool in the ED setting?

## Methodology

Adhering to traditional ethnographic principles, our study placed emphasis on prolonged participant engagement and the establishment of the reliability and validity of findings through rigorous data collection, analysis, and interpretation processes (Lincoln & Guba, 1985). To enhance methodological rigour of our research, we employed multiple styles of data collection (Rashid et al., 2015). Accordingly, primary data were drawn from fieldwork observation notes, video-recordings, and group sessions’ audio-recordings. With this approach, we were able to identify similar characteristics across various data sets and adequately interpret and validate the findings and subsequent conclusions (Thurmond, 2001).

### Study Characteristics

Our study was part of a wider research focused on development and evaluation of a web-based application supporting appropriate antibiotic prescribing by junior doctors in EDs (Hamill et al., 2019). We employed VRE to enhance our understanding of the factors that influence antibiotic prescribing in practice, and the actual use of electronic support tools in healthcare settings. This work was conducted as part of the first author’s doctoral research. The study obtained full approval from the ethical committees of the Gold Coast Hospital and Griffith University (HREC/2019/QGC/57019; GU 2020/965).

Our research team consisted of a diverse group of professionals, bringing together clinicians and researchers with expertise in applied clinical research, antibiotic stewardship research, implementation science, and the use of video-reflexive methods. All team members were affiliated with the same healthcare organisation but worked across different departments including EDs, infection control, pharmacy, medical education, and research. The team consisted of both experienced senior clinicians and junior clinical researchers, with several members holding external appointments with various academic institutions, further enriching the diversity of expertise within the team.

The research was conducted from November 2019 to March 2021, across two EDs located within the same health service in Queensland, Australia. With 83 treatment spaces, the larger ED is a major trauma centre consisting of various sections including triage, resuscitation, acute (non-resuscitation, non-ambulatory patients), minor injuries, children’s emergency, and short stay area. The smaller ED contains 38 beds, enabling care for both adult and paediatric patients. Teams working in the ED consist of individuals with diverse clinical experience including senior and junior doctors, nurses, nurse practitioners, nurse unit managers, occupational therapists, physiotherapists, social workers, and administrative staff. With over 185,000 presentations recorded annually, they are among the busiest EDs in the state.

### Participant Recruitment

To minimise impact on care delivery in EDs, we used existing departmental meetings and training sessions to actively promote our study. These presentations served as a platform to engage with clinicians working in EDs, providing the opportunities to explain the use of video cameras, the purpose of recording, and the ways to participate. As our study focused on decision-making, it was essential that potential participants had the authority to prescribe antibiotic medication. Purposeful sampling was used to recruit participants, with efforts made to gather maximum diversity and a range of clinical expertise and experiences. Clinicians who agreed to be video recorded provided written informed consent and were recruited.

Engaging the VRE concept of care, verbal consent was additionally sought from all participating clinicians on the day of filming. Specific measures were used to ensure awareness of the ongoing filming, which included the distribution of informative emails about VRE to all ED staff, display of ‘recording in progress’ signs during video-recording, and seeking verbal consent from other senior clinicians working in ED space who were present, but not directly involved in the study.

### Video-Ethnographic Observations

The first author conducted initial fieldwork observations to develop better understanding of the ED workflow, team dynamics, and potential camera placement locations. Observations done prior to implementation of video-ethnography helped inform pragmatic decisions on which aspects of clinical decision-making should be captured on video, optimal timing for filming, and potential quality and adequacy of the resulting video data. We further considered the rotational and unpredictable context of the ED and conducted filming during day and early evening, weekday, and weekend shifts. Rather than predefining a specific sample size, we prioritised capturing data with depth and significance that could foster meaningful analysis and initiate discussion during group reflexive sessions (Braun & Clarke, 2021; Vasileiou et al., 2018). To capture diverse ‘decision-making moments’, various interactions were recorded – including informal conversations between clinicians, formal team discussions during clinical handovers, clinicians using electronic support tools or completing clinical documentation, as well as telephone conversations between ED clinicians and staff in other departments.

Video-ethnographic data were collected over a two-month period in 2020. Despite the outbreak of COVID-19 pandemic and initial suspension of most research activities, our study eventually continued with some adjustments due to the minimal local transmission rates and absence of mask mandates at the time. The video observations took place in the ambulatory emergency care area, a setting where patients requiring antibiotics were likely to be treated. To ensure comprehensive coverage, three Go-Pro® HERO-8 cameras were intentionally positioned at different locations to capture a 360-degree view of centralised work-hub station, where team conversations commonly took place. Filming in this location was deemed to be the least disruptive. Throughout the video observations, the first author maintained a continuous presence in the ED space, engaging in informal conversations with clinicians and documenting observations and insights.

### Video-Reflexive Group Sessions

Following video-ethnographic observations, clinicians working in EDs were invited to participate in researcher-facilitated reflexive sessions. Mirroring participant recruitment approach, we used existing departmental meetings to deliver group sessions and minimise impact on patient care delivery. Group reflexive sessions were delivered in the final stages of our study, over a three-month period in 2021. The research team worked collaboratively to select video material representing typical antibiotic prescribing practices. Selected video snippets were played back to clinicians with the intent of encouraging discussion and generating opportunities for collective learning. Video snippets typically lasted between 2 and 5 minutes. Facilitated sessions were audio-recorded and included in the study analysis. Adobe Premier Pro software was used for video data editing, while Microsoft Office Excel and Word applications were used for managing the textual data.

Due to the absence of published guidelines on the selection of video snippets for VRE studies, a structured approach was used to determine the video content for the reflexive sessions. Initially, the first author reviewed all the video data, selecting clips that depicted antibiotic prescribing conversations. Three members of the research team then collectively reviewed all ‘decision-making moments’ and offered guidance on what was considered representative of everyday practice. The selection process considered observable aspects of clinical conversations in line with the research focus, the perceived value and uniqueness of the content, and the potential of footage to generate team discussion. Five clips were ultimately selected and shown in reflexive sessions.

### Data Analysis

Collected video- and audio-recordings were transcribed verbatim. Using inductive approach, we undertook reflective thematic analysis following Braun and Clark’s systematic six-step process to interpret data, develop themes, and report the findings (Braun & Clarke, 2006, 2022). This approach was deemed highly suitable due to its theoretical flexibility and its capacity to delve into the dataset, allowing for the exploration and construction of broader patterns. After coding the data and generating initial themes, we conducted regular meetings to further refine concepts and construct final themes. Additionally, we selected exemplar quotes for each of the identified themes and reported them in the Findings section.

## Findings

A total of 24 clinicians participated in video-ethnographic observations, producing 56 hours of video footage. From this material, 29 clinical conversations regarding antibiotic treatments of 21 patients were deemed most relevant for analysis. Identified conversations varied in length, with majority falling within 3- to 5-minute range. Among the patients who sought treatment, half were already taking oral antibiotics prior to presenting to the ED, and significant proportion had underlying health conditions that influenced treatment considerations. Following careful selection of video snippets, three group-reflective sessions were delivered with 34 clinicians in attendance. An overview of fieldwork data is presented in Table 2.

**Table 2.** Summary of Fieldwork.

Time period	Field site	Data types	Activities and data characteristics
Nov 2019–Jan 2020 (interruption due to COVID-19) and Apr 2020–Sep 2020	Large ED located within the tertiary-level university hospital and smaller ED servicing district hospital within the same health service	Ethnographic observations and researcher field notes relating to informal conversations with clinicians working in ED	*Study preparation and fieldwork observations* ⋅ Two 2-hour fieldwork observations by the primary researcher to each of the participating ED sites ⋅ One presentation about the study to large ED audience at a monthly educational forum ⋅ Five short information sessions held at ED weekly meetings, directed at new clinicians commencing in ED
Oct 2020–Nov 2020	As above	Video-recordings of clinicians engaging in conversations or discussions about prescribing antibiotic treatments for patients under their care	*Video-ethnography* ⋅ Over 56 hrs of video recorded during 18 site visits ⋅ 24 clinicians filmed discussing antibiotic use in patient care ⋅ 29 clinical conversations about 21 patients being observed ⋅ Majority of clips 3- to 5-minute long, with shortest being 1 min 29 sec and longest being 13 min 09 sec ⋅ 27/29 conversations interrupted on multiple occasions by other clinicians, support staff, patients, mobile phones, and overhead announcement systems ⋅ 2 major network interruptions occurred affecting access to integrated electronic medical record system *Patient characteristics(n = 21)* ⋅ 11/21 receiving oral antibiotics prior to presenting to ED ⋅ 12/21 patients with multiple comorbidities, e.g., immunocompromised, chronic kidney disease, and diabetes mellitus ⋅ Presenting conditions: 10/21 cellulitis/wound infections; 6/21 urinary tract infections; 3/21 otitis media; 2/21 others
Jan 2021–Mar 2021	Other hospital settings (various meeting rooms located within the hospital, outside ED department)	Audio-recordings of facilitated group reflexive sessions	*Video-reflexivity* ⋅ Three facilitated group reflexive sessions delivered ⋅ A total of 34 clinicians participated ⋅ Session length 30–45 min ⋅ Seven clips selected by the research team for viewing at the group sessions ⋅ Clips generally 2–3 min in length

Four main themes were identified from the transcribed video and audio data relating to antibiotic prescribing: (i) importance of clinical judgement, (ii) usability of prescribing guidelines, (iii) managing patient expectations, and (iv) context-dependent disruptions (see Table 3). The potential acceptability of the VRE method as an improvement tool in the ED setting was ascertained primarily from the researchers’ field notes and reflexive sessions.

**Table 3.** Main Themes, Sub-Themes, and Exemplar Quotes.

Theme	Subtheme	Exemplar quotes
Importance of clinical judgement	The reliance on clinical experience	“Female with flank pain, history of fevers, nausea, temperature of 38, and background history of kidney stones. So when you hear something like that you need to think down the likes of is it kidney stones, is it infection or is it both … if the patient is febrile, in pain, and tender, you need to think about palletise rise and start to treat empirically … Usually the way we do it is shot of Gentamicin and amoxicillin IV, it’s a single shot gem … you can reduce that dose, and switch to oral antibiotics given but initially you start treating. In emergency, you start treating, that’s IV straight away.” (VRE10) “But usually once they have been seen by one of the surgeons, we get them to review them before we give them antibiotics. So normally for antibiotics you would look up the ETG but I do this all the time, so I don’t need to ... usually for the skin infections it’s fluclox. Sometimes with plastics and orthopaedics because of wounds over bones and structures and stuff like that, they like to sometimes change it up and do cefazolin.” (VRE2) “So, I didn’t look at ETG, I just prescribed what I normally prescribe, as she is uncomplicated, sound patient with no drug allergies, she has not had prior UTIs. So that didn’t make me think, oh, I would need to go and look at her pervious sensitivities, and see if she grows weird bugs that would require different … And from my memory, I might be wrong, but from memory ETGs says Trimethoprim is a good first choice for someone with uncomplicated UTI.” (VRE19) “I was thinking that we should admit him in short stay. Like if I was at Robina in fast track and most of the time you are there by yourself, there is not as many senior clinicians around. So, if he was at Robina, I would have gone down the IV pathway and straight to short stay. But he is being discharged on orals.” (VRE4) “You need to kind of use you clinical judgment, because you need to know what a sick patient looks like … I’m a doctor but I worded for 5 years as an ICU nurse so I have seen plenty of very sick people. They look different, they are uncomfortable, they are in pain, they are not moving, they are not just sitting texting on their phone, they are genuinely freaked out, so yeah … And pain is one of the biggest things. If I touch this particular patient in the flank area, and literally tap very gently, she would jump of the bed screaming, and then you know something is wrong.” (VRE10)
	Clinicians’ collaboration in prescribing decision-making	“Initially I did not think that he would need antibiotics, but the consultant said – oh it’s a dirty wound, quite deep leg laceration from a rusty nail – so, he decided, so we then decided to put him on antibiotics.” (VRE5) “It’s one of those decisions where you can’t know it all, and we couldn’t get lumbar puncture results for about 7 hrs, but yes there was definitely a lot of uncertainty. I was definitely glad that I was supported by the team who knew where to go. There was a lot of conflicting factors, you couldn’t rule out much, you couldn’t narrow it down … The patent could not provide much information because she was confused … which was probably another symptom, and the fact that she had multiple seizures and she had infection … or even just behavioural issues too ... So, yes, it was quite a difficult case.” (VRE18) “I would always advocate that if you are an intern or JHO, to always discuss it with someone who is senior. All patients if you are an intern or JHO must be discussed with a registrar or a consultant. So everything, no matter what. It’s for learning but also patient safety. Before they [JHOs] initiate a treatment, they say, hey this is what I’ve done, and this is what I would like to do. So rather than saying what shall I do, they say, this is what I think we should do.” (VRE1) “Patient is not sick enough to need IV treatment, but she failed to respond adequately to cefazolin in the past … fuyclox is probably just as efficacious … So a junior doctor has seen her prior to me, and I was reviewing the case with him to make a decision and the question is whether we just treat it for failure only and see what response we get v just giving her an antibiotic as well. Because of the lack of warmth and tenderness it was less likely to be infected. We sent the photographs to the last reviewing doctor and we have a treatment plan and they will review her in 48 hrs … During business hours we can always phone them[GP] … we took some photographs with the patient’s permission and sent it to GP to see … what do you think of this compared to when you saw her last … so continuity of care is these cases is really useful.” (VRE11)
	Managing patient risk	“He is systemically well but he does have diabetes type 1, he is on insulin, so given that fact, I opted for IV antibiotics, as he is a higher risk.” (VRE28) “This lady has been treated for lower limb cellulites up until a week ago, she has been treated with cephalexin. She has come back with damages, weeping changes, panful, tight – has heart failure, it wasn’t hot or cellulitis but it has changed since presenting last … I’m not convinced there is additional infection, but it’s progressively gotten worse and she is immunosuppressed, with diabetes and rheumatoid arthritis.” (VRE7) “The problem is she has already been on the antibiotics, and she is a diabetic.” (VRE2) “So if anything is penetrating, realistically, it’s. You can’t clean to the base of it, so you got something that’s penetrating deep … it’s one of those things where you can see what happens. He is very unlikely to get sick … if we discharge him now and he came back with cellulitis, we can treat that, it’s not going to get septic. If we had given him a staph dose and send him home early, it would prevent him from coming back? Maybe … you know.” (VRE19)
	Managing unknown	“So this is the advice for empirical therapy while we are waiting for the results of the investigation … Unfortunately in this case, the investigations were never sent in [by the GP] the first place.” (VRE16) “So we were postponing the antibiotics because we were also waiting on the CT to see if there was another cause relating to her symptoms … But yeah, once she spiked a fever, we pretty much went down the fever infected pathway and we did some more blood cultures and at that point we new probably we need to do a lumbar puncture.” (VRE24) “Sometimes you don’t even know a diagnosis and you have to start treating, for example if the patient is septic we start treating and then as it unravels you adjust the treatment.” (VRE15)
Usability of prescribing guidelines	Prescribing guidelines adherence	*Researcher*: “So do you at this point know the dosage and duration you will give him?” *Clinician*: “So usually, 300 for a 7 days course …” *Researcher*: “So is this based on your previous experience?” *Clinician*: “I would also check ETGs.” *Researcher*: “I guess it’s not always a clear cut?” *Clinician*: “Yes, especially for a geriatric patient with comorbidities … But again, he is not septic, he is not febrile [looks trough ETG] … Oh, it says 3 days …” *Researcher*: “So 300 mg, 3 days orally is general recommendation? So does it give you what to do for recently treated pt or how to modify treatment, or do you make that yourself based on previous experience?” *Clinician*: “So, it’s essentially a clinical decision, so it’s a guideline, always, and it’s at physician’s discretion. So we will treat him for 3 days and then review, as he might have sensitivities to antibiotics so we can switch, and his GP can check for him.” (VRE23)
	Lack of effective use of electronic guidelines	“So ETG is on the network, and you can log in, but sometimes you have to scroll quite a bit to get to the bit you actually want and sometimes the suggesting on how to score someone’s severity is not the most clear. It should be succinct but give you some clear guidelines.” (VRE1) “But without everything in one place ... so, it’s not useful for me to look at, I need everything in one place, otherwise it’s too many things going on.” (VRE22) “Back home we also have an app, something similar, but it has loads more guidelines for literally everything. Like you will go down and it will have the scoring, the guidelines, whatever – it’s like ETG but more clear and on your phone as well – it was called micro guide. I have used ETG couple of times, but I don’t think it covers everything.” (VRE4) “So my go to for antibiotics is go there if I am not sure, but is think some of the places are a bit wordy. If they just said look, this is the scoring system, these are the points, if it’s this use this, if it’s this, use this. There is quite a bit of practitioner-to-practitioner difference … And in general very broad-spectrum antibiotic as opposed to back home they were very strict on what you could and could not do, and certain things would need approval from microbiology. I’ve noticed it’s definitely a bit more loose here.” (VRE2)
	Clinicians’ use of workarounds	“Spoke to [Senior registrar] regarding usability of ETGs and how it works in practice, and he offered to demonstrate ‘the process’ of searching for a suitable antibiotic for a rare critical condition. The access to site in not working – the system then displays a message it doesn’t trust the site … ‘it’s not usually this slow’ he remarks, as he waits for few moments … then jokes ‘well this is taking too long, so I will switch to our illegally installed google chrome.’ Few other clinicians join in. I ‘I don’t know, I restarted this computer before I started today and ETG was working’ … I ask ‘if this was a real case, what would you normally do if it’s not working? As a senior clinician?’ … ‘just pick some antibiotics from my brain” he states, elaborating further about the need to start treating immediately, selecting a broad range antibiotic and then bringing other experienced clinicians in to help … overall, there is a great sense of cheerfulness and team unity on the floor as staff casually interact and carry on chatting about mundane things.” (Observation note 13)
Managing patient expectations	The role of patient education in managing expectations	*Clinician*: “Mum is a bit worried, and she didn’t like the idea of taking the antibiotics, but I think is the right choice.” *Researcher*: “Did she express that?” *Clinician*: “Yes she did and she was upset that her GP didn’t explain it to her, she said it was just quick, she just wrote the script, gave him the antibiotics and said to take that, and that was the end of the story” ….. “So I went to [dr] who was one of my mentors and he said lets up the dose of the antibiotics so we upped the dose. I explained that to mum, and she went ok, lets continue the same treatment that seems to be targeted for that infection and then I’ll follow up with the GP in a couple of days' time.” (VRE 21) “So there wasn’t much of the education for the patient about the treatment and I noticed that once I explained thing to her she felt very comfortable with that.” (VRE27) *Clinician*: “So, I have given her the antibiotics with the advice to follow up with her GP, any symptoms, they might need to consider some renal track imaging or something like that. I’ve given her some return advice [printed information sheet] on tracking vomiting, fevers, things like that and if she needs intravenous therapy. But at the moment I think we will try this, there is no other explanation for it to have a flank pain. There is no history of trauma or anything like that.” *Researcher*: “Do you think that the patient happy with diagnosis, she wasn’t expecting anything more?” *Clinician*: “No … she was happy with the plan and the follow up for the duration.” (VRE16)
	The impact of patient requests	*Senior clinician:* “What we can do is if you have another look over her, get in touch with her GP, and discuss with the GP whether they are happy to continue treating … she is actually happy to continue with taking antibiotics at home…” *Intern*: “Ok, no worries.” *Senior clinician:* “The only other option is then some IV antibiotics in short stay, but her ESR won’t change that quickly, so the minimum is two weeks … so, yes that’s the plan. She is actually quite happy to go home if her blood tests are better, that’s the only reason we’re adding this test.” (VRE 8) *Clinician*: “For example, antibiotic resistance – so this lady, man, very much has shingles on his buttocks … But mum is absolutely certain that it is not shingles, because she had shingles and it doesn’t feel the same to her, and she thinks it’s cellulitis. And he has had multiple abscesses on his buttocks. I do not think that it is infected, but the mum is very adamant that it is infected and that she won’t take the antivirals without the antibiotic.” *Researcher*: “She insists?” *Clinician*: “I’m not going to chunk up the fight. So someone who has come in with something very straight forward, and they are wanting this, and you know if you don’t do what they want then …” (VRE20)
Context-dependent disruptions	Types of disruptions	“Throughout the two-month recording period, my respect for the work performed by ED clinicians grew immensely. The ED environment is characterized by high pressure and rapid change, as evidenced by sudden system failures, overcrowding, limited admission beds, staff shortages, and changing patient flow management due to COVID-19 protocols. In addition to these challenges, clinicians must also manage a constant influx of patients with complex comorbidities whose conditions can deteriorate quickly. The ED is a noisy and crowded space, where privacy and personal space are limited, and clinicians are frequently interrupted by phone calls, emails, and colleagues seeking or passing on information. From all the observed interactions that were recorded, only a few were able to complete their discussions without some form of interruption.” (Observation note 29)
	The impact of constant disruptions	“ I didn’t realise how busy it was … they have to constantly make decisions … it’s such a decision fatigue, it’s hard … but then it’s always busy in there, it never stops.” (Group reflexive session 2) “It’s just interesting to observe how busy clinicians are, the last video looks like it was a particularly busy day … She is speaking with one clinician, another one is waiting next to her, it looks like a nurse passing by is telling her something too ... her phone is ringing in her hand … and then there are patients sitting in visitor chairs, we don’t normally have them treated there – it looks like it was a particularly bad day.” (Group reflexive session 3)

### Theme 1: Importance of Clinical Judgement

All the clinicians in our study demonstrated a strong reliance on clinical experience in making treatment decisions. For instance, describing the importance of clinical judgment to identify sick patients, one clinician stated,

You need to kind of use your clinical judgment, because you need to know what a sick patient looks like … I have seen plenty of very sick people. They look different, they are uncomfortable, they are in pain, they are not moving. (VRE10)

Similarly, a junior clinician shared their approach to treating a patient with flank pain and a history of fevers: “So when you hear something like that you need to think down the likes of, is it kidney stones, is it infection or is it both … in emergency, you start treating, that’s IV straight away” (VRE10). While discussing a patient’s treatment with a student, a senior doctor likewise explained,

So, I didn’t look at ETG [electronic therapeutic guidelines], I just prescribed what I normally prescribe ... And from my memory, I might be wrong, but from memory ETGs says Trimethoprim is a good first choice for someone with uncomplicated UTI. (VRE19)

Overall, clinicians relied on their experience and judgment when making treatment choices, highlighting the significance of clinical expertise in the antibiotic prescribing practices within the ED setting.

Frequent clinical collaboration was another factor readily observable on video. Junior clinicians often consulted senior colleagues, seeking their guidance and expertise when faced with complex clinical cases. While clinicians mostly prescribed antibiotics in accordance with relevant guidelines, challenges of choosing appropriate treatment in the face of clinical uncertainty were evident. In one instance, a clinician stated,

Initially, I did not think that he would need antibiotics, but the consultant said – oh it’s a dirty wound, quite deep leg laceration from a rusty nail … so we then decided to put him on antibiotics. (VRE5)

Another junior clinician reflected,

If I were by myself at the [smaller ED] I would not be confident discharging this patient on oral antibiotics, I would be more careful. But because [senior clinician] was here and he had reviewed them, I feel much better in making this decision. (VRE4)

The input and guidance from senior clinicians were particularly valued during challenging patient presentations, with discussion seen as essential for patients’ safety and ensuring well-informed decision-making. In one participant’s words,

It’s one of those decisions where you can’t know it all, and we couldn’t get lumbar puncture results for about 7 hrs, but yes there was definitely a lot of uncertainty. I was definitely glad that I was supported by the team who knew where to go. There was a lot of conflicting factors, you couldn’t rule out much, you couldn’t narrow it down … So, yes, it was quite a difficult case. (VRE18)

Reliance on collaborative efforts and the value of having a supportive team during challenging cases were seen as essential, particularly for junior clinicians with limited experience.

Risk aversion at times influenced the selection of antibiotics administration route. For example, a senior clinician spoke of a quick, straightforward decision to admit a patient:

He is systemically well but has type 1 diabetes, on insulin, so a high risk of acquiring further complications, large area of cellulitis, four days … if he didn’t have diabetes, he would then have oral tablets and we would send him home, but because of this, he is more prone to complications. (VRE25)

Another clinician observed, ‘This lady has been treated for lower limb cellulitis up until a week ago … She has come back with damages, weeping changes, painful, tight – has heart failure … I’m not convinced there is additional infection, but its progressively gotten worse and she is immunosuppressed, with diabetes and rheumatoid arthritis’ (VRE15). Typically, having to actively manage compounded risks such as patients’ comorbidities or the possibility of missing underlying infections motivated clinicians to act with abundance of caution.

Managing the uncertainty of patients’ diagnosis was an additional factor affecting antibiotic prescribing decisions. In cases where a definitive diagnosis was elusive, clinicians resorted to empirical therapy while awaiting investigation results. As one clinician explained,

So we were postponing the antibiotics because we were also waiting on the CT to see if there was another cause relating to her symptoms … But yeah, once she spiked a fever, we pretty much went down the fever-infection pathway. (VRE24)

At times, there was a need for immediate intervention to address potentially life-threatening conditions, despite the absence of confirmed diagnosis. A senior clinician observed, “Sometimes you don’t even know a diagnosis and you have to start treating, for example, if the patient is septic we start treating and then as it unravels you adjust the treatment” (VRE15). These experiences highlight the challenging nature of diagnostic uncertainty in the ED and the critical role of adaptive decision-making in providing timely and appropriate treatment.

### Theme 2: Usability of Prescribing Guidelines

While video observations showed that clinicians routinely prescribed antibiotics in accordance with the clinical guidelines that were accessed electronically, diverse opinions emerged regarding their usability in practice. Positive aspects included the comprehensiveness of prescribing guidelines, their coverage of various conditions, and accessibility through multiple electronic platforms. One clinician observed,

So many people are using their phones now … I have a section in here for all my medical apps. All my guidelines, kids’ guidelines, conversion for drugs, I even have the app I used in the medical school … I think something as an app is quite easy for someone to just look up … it’s really, really useful. (VRE1)

Another clinician confirmed, “I use ETGs all the time, it’s my go to for everything” (VRE4). However, others expressed contrasting views about the difficulties in quickly locating specific instructions, the need to consult multiple sources for treatment information, and the lack of concise presentation of key data. A clinician mentioned the challenges of navigating through the ETG, stating,

I don’t think it’s quite straightforward and succinct ... you have to scroll quite a lot to get to the bit you actually want and sometimes the bit that is suggesting how to score someone’s severity is not the most clear, so you have to check elsewhere. (VRE28)

Another junior doctor remarked, “it’s not useful for me to look at, I need everything in one place, otherwise it’s too many things going on” (VRE22).

Occasionally, clinicians resorted to workarounds to locate needed information quickly, particularly when experiencing procedural barriers such as computer system disruptions. During one fieldwork observation, a senior clinician was demonstrating a search process for a suitable antibiotic for a rare condition, when a system interruption occurred:

The access to site in not working – the system then displays a message it doesn’t trust the site … It’s not usually this slow – he remarks, as he waits for few moments, then jokes – well this is taking too long, so I will switch to our “illegally” installed google chrome. (Observation note 13)

Despite the differences in opinion about the usefulness of ETG, most clinicians overall acknowledged their value as an initial reference point in antibiotic treatment considerations that was complemented by further discussions with colleagues in the ED.

### Theme 3: Managing Patient Expectations

Managing patient expectations was another major theme that highlighted how patients’ requests and health literateness impacted treatment plans. In certain instances, patients played an active role regarding their treatments, expressing their preferences or wishes. This sometimes led to individuals’ requests taking precedence over clinicians’ judgement or deviations from established guidelines. Such actions presented both opportunities and challenges in practice. One clinician described a situation where a patient insisted on receiving antibiotics despite their professional judgment, stating “I do not think it is infected, but she is very adamant that it is infected and that she won’t take antivirals without antibiotics, so … I’m not going to start up a fight” (VRE20). On the other hand, a team of clinicians discussed an elderly patient who expressed a preference for continuing oral antibiotics at home, stating

We were going to keep her in short stay but she is actually quite happy to go home and continue with orals if her blood tests are better … and she wants us to call her GP who finishes at 5, I’m sure we can accommodate that. (VRE8)

The provision of sufficient education regarding intended treatment contributed to the management of patient expectations. Several clinicians observed that patients at times prematurely presented to the ED seeking treatments, either due to poor health literacy or perceived non-effectiveness of antibiotics. One clinician stressed the importance of educating a concerned parent of a paediatric patient, stating

mum is a bit worried, and she didn’t like the idea of taking the antibiotics … she was upset that her GP didn’t explain it to her … just wrote the script, gave him the antibiotics and that was the end of the story. I explained that to mum, and she went ok, lets continue the same treatment that seems to be targeted for that infection. (VRE21)

Likewise, a clinician noted a positive impact of education, stating “So there wasn’t much of the education for the patient about her treatment, and I noticed that once I explained things to her, she felt very comfortable with that” (VRE27). Clinicians who set aside time to explain intended treatments and address patient concerns typically exhibited more effective management of patient expectations.

### Theme 4: Context-Dependent Disruptions

Constant disruptions in the ED were another major theme identified in our study. As was revealed through group reflexive sessions, video observations realistically captured busy ED conditions – all the noise, interruptions, and constant distractions. In one reflexive session, junior clinicians were particularly interested in unpacking the aspects of weighty responsibility of a senior clinician leading a team on the day. The footage showcased simultaneous conversations occurring with multiple team members and high demand for senior clinicians’ attention. One clinician observed, “I didn’t realise how busy it was … they have to constantly make decisions … it’s such a decision fatigue, it’s hard … but then it’s always busy in there, it never stops” (Group session 2). In another session, clinicians focused on busyness of the ED space, stating

The last video looks like it was a particularly busy day … She is speaking with one clinician, another one is waiting next to her, it looks like a nurse passing by is telling her something too … her phone is ringing in her hand … and then there are patients sitting in visitor chairs, we don’t normally have them treated there-it looks like it was a particularly bad day. (Group session 3)

Upon reflection, clinicians recognised the impact of these factors on driving the adoption of shortcuts in decision-making.

Video observations further captured the types of disruptions clinicians encountered. Disruptions included ringing of phones, beeping of various medical instruments and alarms, overhead speaker announcements, and, on two occasions, complete loss of critical electronic systems, that required the use of handwritten notes and visual communication through whiteboards to manage patient flow. Fieldwork observation notes reflected on this:

The ED environment is characterized by high pressure and rapid change, as evidenced by sudden system failures, overcrowding, limited admission beds, staff shortages, and changing patient flow management due to COVID-19 protocols. Furthermore, clinicians also manage a constant influx of patients with complex comorbidities whose conditions can deteriorate quickly. The ED is a noisy and crowded space, where privacy and personal space are limited, and clinicians are frequently interrupted by phone calls, emails, and colleagues seeking or passing on information. From all the observations to date, only a few were able to complete their discussions without some form of interruption. (Observation note 29)

Overall, video observations and reflexive sessions provided valuable insights into the context-dependent disruptions experienced in the ED. Clinicians acknowledged the demanding nature of their work and highlighted the impact of constant disruptions on their decision-making processes.

## Utility of the VRE as an Improvement Tool

The acceptability of VRE as an improvement tool in the ED setting was likewise examined in this study. Overall, the perceptions of clinicians regarding the use of VRE varied, with some viewing it as a positive approach. However, most expressed indifference towards VRE, while only one clinician specifically declined to participate in the video-recording process. Clinicians who perceived VRE as a positive tool recognised its potential to provide valuable insights into their own antibiotic prescribing practices and to visualise complexity of the ED space. These clinicians acknowledged the benefits of observing interactions and communication patterns captured in the video-recordings. They viewed VRE as an opportunity for improvement and learning, enabling them to identify areas for potential enhancements in patient care delivery.

On the other hand, most clinicians expressed indifference towards VRE during reflexive sessions. They did not convey positive or negative opinions about the use of video in the ED context. For these clinicians, VRE did not significantly impact their daily routines or perceptions of their own practices. This indifference may be attributed to factors such as time constraints, workload pressures, or a lack of familiarity with the potential benefits that VRE research can offer in terms of practice improvements. Only one senior clinician held strong negative views on video observations and declined to participate. Their decision not to be video-recorded highlights the importance of considering individual preferences and comfort levels when implementing VRE research in healthcare practice. Respecting the autonomy and choices of clinicians is crucial to ensure their potential engagement and willingness to participate in future initiatives.

## Discussion

The findings of this study highlight several factors affecting antibiotic treatment choices and provide valuable insights into the challenges and opportunities associated with implementing VRE in the ED setting.

The *importance of clinical judgment* in the decision-making process was emphasised in our study. Clinicians underlined the significance of relying on their experience and the expertise of more senior colleagues when making treatment decisions. They placed value on tailored patient care and the recognition that guidelines alone may not always capture the nuances of individual cases. Challenges clinicians faced in antibiotic treatment prescribing were similar to those reported in the literature (Krockow et al., 2019). They relate to the fact that treatment decisions in practice are closely linked to striking a balance between delivering appropriate care with necessary trade-offs. Therefore, even when a decision may appear to deviate from the guidelines, it still possesses identifiable logic and relevance to clinicians making treatment choices.

The *usability of electronic guidelines* in the fast-paced ED setting was another important theme. While clinicians appreciated the ease of access and comprehensive nature of the guidelines, they found them challenging to navigate at times. In contrast to a recent scoping review that found low actual use of technology in clinical decision-making, our study revealed a regular and active use of technology devices to support antibiotic prescribing decisions (Jun et al., 2018). However, despite the frequent reference to electronic guidelines, clinicians still preferred accessing information that was quick, easy, and immediately available, similar to research findings from other studies (Ly, 2021). Prescribing guidelines were generally considered a useful resource; nevertheless, clinicians often relied on their clinical judgment to deviate from the guidelines when necessary, considering factors such as patient presentation, comorbidities, and clinical uncertainty.

*Managing patient expectations* was another critical factor in antibiotic prescribing. Our study revealed instances where patients’ requests or preferences influenced treatment decisions, sometimes deviating from clinical guidelines. While accommodating such requests can contribute to patient satisfaction, it also poses challenges to antibiotic stewardship practices. A recent survey on patients’ expectation around antibiotic medication in EDs found that clinicians were likely to prescribe antibiotics if they believed patients expected them, or directly asked for them, regardless of clinical judgment to the contrary (Broniatowski et al., 2015). We found that the decisions to prescribe antibiotic treatment were complex and dependent on more than just the clinical factors alone. At times, clinicians chose perceived safe options when prescribing antibiotics, acting with an (over)abundance of caution, due to concerns about occult infection, or atypical presentation deemed a higher risk. Categorising them as ‘compelling motivations’, scholars argue that such instances fundamentally shape clinicians’ behaviour and subsequent choices on medication prescribing (Szymczak, 2018).

The *context-dependent disruptions* experienced in the ED were also a significant theme of our study. Clinicians faced numerous challenges, such as noise, interruptions, and constant distractions, which impacted communication, decision-making, and workflow. The high-pressure environment coupled with time restraints, resource limitations, and technological failures further complicated the delivery of patient care. Disruptions in the ED are not a new phenomenon, with researchers pointing out that these conditions shape the way clinicians think, behave, and go about doing their work (Szymczak, 2019).They also affect the success of new interventions as they influence how clinicians perceive workplace challenges and how they go about accepting and managing change.

Fittingly, major themes identified in our study broadly align with what constitutes evidence-based practice (EBP). The relationship between the three pillars, namely, clinical expertise, research evidence, and patient preferences, was explained by the movement’s pioneer David Sackett as ‘integration of clinical expertise, acquired through experience and clinical practice, with best available clinical evidence, in making decisions about the care of individual patients’ (Sackett et al., 1996). Sackett went on to argue that neither one is enough on its own because “even excellent external evidence may be inapplicable to, or inappropriate for an individual patient, however, without current best evidence, practice risks becoming rapidly out of date, to the detriment of patients” (p. 72). Our findings indicate that context-specific factors such as constant disruptions also influence delivery of patient care and may unfavourably contribute to clinicians’ cognitive burden and decision fatigue. This underscores the need for development of strategies that optimise communication, minimise distractions, and enhance workflow efficiency to support clinicians in providing high-quality care amidst challenging conditions.

Regarding the feasibility of VRE in the ED setting, our study identified factors that can hinder or enhance its use. Implementing this research approach required significant time investment in familiarising clinicians with video-ethnography and video-reflexivity, establishing trust, navigating ethical considerations, and addressing clinicians’ concerns. Overall, video footage of *in-situ* work practice provided clinicians with increased opportunity for collective discussion. At reflexive sessions, junior clinicians identified aspects of their work practices that were done well, such as locating and applying relevant guidelines, formulating effective treatment plans, and communicating in a clear and concise manner. Clinicians repeatedly commented on ‘hustle and bustle’ and ‘organised chaos’ of the ED environment. Some clinicians supported the video-observational approach as a useful way of ‘seeing’ individuals’ habitual practices and the complexity of clinical decision-making, stating that dynamics and complexity of clinical interactions have not been apparent to them previously. Framed this way, VRE may be useful for ‘seeing’ and facilitating rapid assessments of unfolding care, but may be difficult to sustain long term due to the operating nature of ED teams – which are demand driven and continuously changing, with limited time for continuous team reflection.

## Limitations

Our study had several limitations. Firstly, the onset of COVID-19 and hospital restrictions impacted our research activities, resulting in delays in video-observation collection and delivery of facilitated group sessions. The location where our video observations took place would typically treat patients presenting with upper respiratory tract infections with fever and cough. However, these patients were triaged in a dedicated COVID-19 space, separate from the main ED. Consequently, our access to cases that are commonly targeted for antibiotic stewardship improvements was limited. Similarly, redeployments of medical staff into other critical areas reduced availability of certain individuals to participate consistently from start to finish.

Secondly, our study was conducted in two EDs with a well-established research culture and active engagement of senior clinicians in scholarly and educational activities. As a result, the majority of clinicians were receptive and actively supportive of new research initiatives.

Thirdly, we acknowledge that our research may not be representative of the complex clinical decision-making that occurs in other EDs; nonetheless, our findings provide useful insights into factors that shape clinical experience among clinicians working in similar contexts elsewhere.

## Conclusion

Overall, our study provided a visually rich account of antibiotic prescribing practices in busy ED environment. The findings highlighted ongoing tension between adherence to guidelines and pressing need to use practical knowledge in clinical judgement. Prescribing choices were fundamentally influenced, not just by clinical information from medical guidelines but also by the contextual factors that affected individual and collective day-to-day work practice, which remain poorly described in the literature. Although not immediately measurable, the impact of VRE has been evident in more meaningful dialogue among clinicians around antibiotic prescribing occurring as our study progressed. We have also established that VRE is feasible in the busy ED setting but challenging to implement and sustain without sufficient resources. While this approach may not be suitable for every team environment, VRE can be particularly useful for high preforming clinical teams with a strongly established improvement and research culture who are invested in improving own clinical practice. Future research should focus on exploring implementation strategies and mechanisms that support video-reflexivity integration in the ED setting.
